# Structural Features of the Telomerase RNA Gene in the Naked Mole Rat Heterocephalus glaber

**Published:** 2014

**Authors:** S. A. Evfratov, E. M. Smekalova, A. V. Golovin, N. A. Logvina, M. I. Zvereva, O. A. Dontsova

**Affiliations:** Faculty of Chemistry, Moscow State University, 1-3 Leninskie Gory, Moscow, 119991, Russia; Faculty of Bioengineering and Bioinformatics, Moscow State University, 1-40 Leninskie Gory, Moscow, 119991, Russia; Belozersky Institute of Physicochemical Biology, Moscow State University, 1-40 Leninskie Gory, Moscow, 119991, Russia

**Keywords:** H. glaber, telomerase RNA, bioinformatics, promoter analysis, comparative genomics.

## Abstract

Telomere length, an important feature of life span control, is dependent on the activity of telomerase (a key enzyme of the telomere-length-maintaining system). Telomerase RNA is a component of telomerase and, thus, is crucial for its activity. The structures of telomerase RNA genes and their promoter regions were compared for the long-living naked mole rat and different organisms. Two rare polymorphisms in *Heterocephalus glaber *telomerase RNA (hgTER) were identified: A→G in the first loop of pseudoknot P2b-p3 (an equivalent of 111nt in hTR) and G→A in the scaRNA domain CR7-p8b (an equivalent of 421nt in hTR). Analysis of TER promoter regions allowed us to identify two new transcription factor binding sites. The first one is the ETS family site, which was found to be a conserved element for all the analyzed TER promoters. The second site is unique for the promoter region of TER of the naked mole rat and is a binding site for the SOX17 transcription factor. The absence of one Sp1 site in the TER promoter region of the naked small rat is an additional specific feature of the promoter area of hgTER. Such variation in the hgTER transcription regulation region and hgTER itself could provide increased telomerase activity in stem cells and an extended lifespan to *H. glaber*.

## INTRODUCTION

Several studies have shown that stem cell function is impaired during aging [1-3]. A decrease in stem cell function may contribute to impaired maintenance and the function of some tissue during aging. The role of telomere shortening was identified to be the mechanism contributing to the accumulation of DNA damage in replicative aging of stem cells [4]. Experiments in mTER −/– mice with telomere shortening have provided the experimental evidence that this process can impair the function of somatic and germline stem cells [5]. Telomerase is a key component of the telomerelength- maintaining system and an important contributor to the reduction of replicative senescence in germ and stem cells [6, 7]. Temporary telomerase reactivation in late-generation TERT -deficient mice extends telomeres, reduces DNA damage signaling and associated cellular checkpoint responses, and eliminates degenerative phenotypes across multiple organs, including testes, spleen, intestine, and even neurons [7]. Moreover, temporary telomerase expression in aged normal mice significantly increases the lifespan of mice [8].

Telomerase synthesizes new telomere repeats at the G-strand and thus participates in the compensation for telomere loss during replication [9]. Two components are required for telomerase activity *in vitro*: a reverse transcriptase catalytic subunit (TERT ) and telomerase RN A (TER ) that contains a template for telomere synthesis [10]. TERT was shown to play a role in a number of cellular processes (cell cycle response, oxidative stress, antiapoptotic action, etc. [11]) outside of the telomerase complex. No hTERT was found in most differentiated normal tissues, although a low level of hTERT could be detected in the skin, spleen, stomach and small intestine and a higher level was detected in testes and the endometrium [12]. In contrast, hTR expression was detected in many normal tissues, including testes, ovary, brain, liver, small intestine, thymus, kidney, and prostate, suggesting that telomerase RN A may also have alternative functions [13]. In some cancer cell lines, the level of TERT expression is critical for telomere elongation; however, in case of stem cells in a living organism it is the high level of TER expression that is more important for telomere elongation. Indeed, an analysis of interspecies crosses of TER - and TERT -deficient mice [14] showed that the increase in the gene copy number of TER , but not TERT , is what is critical in telomere elongation. Ectopic expression of hTER caused telomere elongation in bovine blastocysts, whereas co-expression of hTERT and hTER did not result in further increase in the telomere length [15], providing further evidence of the fact that the level of telomerase RN A is critical for telomerase activity and telomere elongation in the cell within the organism.

The genome and transcriptome of the naked mole rat have recently been sequenced [16, 17]. *Heterocephalus glaber*, the naked mole rat (*H. glaber*), has a very high life expectancy among rodents ( > 28 years vs 1.5–7 years in other rodents), high resistance to carcinogenesis and retarded aging [18].

A comparative study of the *H. glaber *genome can help reveal the reasons for the surprisingly long lifespan of this animal. A number of genetic alterations have already been found, which could explain the increase in the DNA repair level, as well as the reduced oxidative damage or reduced replicative senescence [5, 19]. Another reason for the longevity could be the higher level of telomerase activity in *H. glaber *stem cells or telomerase reactivation under a certain type of stimulus. In this study, we have compared the structure of telomerase RN A genes and their promoter regions for the longliving naked mole rat and different organisms with an aim to identify the features that may increase hgTER expression and telomerase activity in stem cells.

## MATERIALS AND METHODS

**Comparison of TER sequences**

To search for the *hgTER *gene of *H. glaber, *we used the whole genome shotgun project entries with IDs: AFSB (GenBank: AFSB00000000.1) and AHKG (Gen- Bank: AHKG00000000.1). We used ClustalW to build an alignment of the sequences of interest with the already well-described telomerase RN A genes. The following reference sequences were used: *Cavia porcellus *TER (GenBank: AF221929.1), *Cavia porcellus *WGS assembly (GenBank: AAKN00000000.2), *Chinchilla chinchilla* TER (GenBank: AF221937.1), *Chinchilla chinchilla* WGS assembly (GenBank: AGCD00000000.1), *Mus musculus *TER (GenBank: NR _001579.1), *Mus musculus* chromosome 3 (GenBank: NC _000069.6), *Rattus norvegicus *TER (GenBank: NR _001567.1), *Rattus norvegicus *chromosome 2 (GenBank: NC _005101.3), human TER (GenBank: NR _001566.1), human chromosome 3 (GenBank: NC _000003.11), *Danio rerio *TER (GenBank: EF569636.1), and chromosome 25 (GenBank: CU 651628.3).

The alignment analysis and the influence of polymorphisms on the secondary structure were performed manually. The sequences of TER s from *Suncus murinus* (GenBank: AF221921), *Geomys breviceps *(GenBank: AF221930), *Microtus ochrogaster *(GenBank: AF221909),* Mus spretus *(GenBank: AY058901), *Mus musculus* (GenBank: AY058900), *Dasyurus hallucatus *(GenBank: AF221919), *Bufo japonicus *(GenBank: AF221913) and* Typhlonectes natans *(GenBank: AF221910) were used to perform a secondary structure analysis.

**Comparison of TER promoter areas**

We searched for promoter regions using the Jaspar database [20], restricting the search to the Jaspar CORE Vertebrate with a 99–100% relative profile threshold. ConSite with a 85–95% TF score cutoff was used for further analysis of the promoter sequences [21]. Relative scores were used as normalized score values for the quantitative evaluation of hit significances [22]. Hit corrections were done manually where necessary. Visualization of multiple alignments was corrected manually

## RESULTS

**Identification of H. glaber TER**

The full *hgTERC *gene (*Heterocefalus glaber *TER ) was identified by local BLAST on the basis of the *H. glaber* genome assembly (WGS record AHKG) and a comparison with multiple alignment data for mammalian TER sequences [23]. The final alignment is available at 93.180.62.254/hgTERC /ESM_1.pdf.

According to phylogenetic data, the closest relatives of *H. glaber *are *Hydrochoerus hydrochaeris*, *Cavia porcellus*,* Chinchilla chinchilla*, and *Myocastor coypus *[18]. TER sequences are known only for *Chinchilla chinchilla* (GenBank: AF221937.1) and *Cavia porcellus *(Gen- Bank: AF221929.1); those were used for further structure comparison. Furthermore, the data for human TER were used for the analysis due to the availability of detailed information about the promoter region and secondary structure of TER .

**Comparison of TER promoter areas**

A 500 nt (from the expected transcription start site) promoter region was used for the analysis, since the major regulatory elements were found in this area for human TER (hTER ) [24]. The web services JASPAR [20] and ConSite [21] were used to search for transcription factor binding sites in the promoter area of *H. glaber*. These tools provide the possibility to analyze any sequence data; they are relatively simple in operation and allow one to deal with large-position weight matrices with optimal results due to the effective job filtering. We used strict filtering of the results for both tools to find the most reliable sites.

This approach reduced the number of predicted transcription factor binding sites in the hTER promoter area compared to the number of sites determined earlier [24]. The elements in the promoter region of TER s from four different species identified in this study are shown at 93.180.62.254/hgTERC /ESM_2.pdf (the full promoter map is available at 93.180.62.254/hgTERC / ESM_3_old.pdf). The elements found in the promoter region of hgTER are as follows: TATA box in the proximity of the transcription start site, NF-Y site with the conserved CC AAT box, three SP1 sites, ELK4 site, and SOX17 binding site.

The ELK4 transcription factor binding site had never been identified for any TER before; it is located approximately 170 nt upstream from the start of the TER coding region. We have found this promoter element for all tested sequences; *p *values were calculated by MAST. The ELK4 score for the *H. glaber *was 14.059 (the relative score 0.9999; *p *= 3.3∙10-6); 11.056 (0.9034, 4.1∙10-5) for *Cavia porcellus*; 12.053 (0.9311, 2.2∙10-5) for* Chinchilla chinchilla*; and 10.398 (0.9396, 5.5∙10-5) for humans, thus meaning a very high probability (> 90%) of ELK4 transcription factor binding site occurrence in the TER promoter area, at least from the bioinformatic point of view.

When performing the search, we found that the ELK4 binding site [25] matrix in the JASPAR database was outdated [25-27] and this led to a false identification for most proteins containing the ET S domain, except for families I and II [28]. Since we are dealing with a very small set of sequences and the difference between the new and old matrices is negligible, we used the old position weight matrix (PWM).

Multiple alignments showed that a ET S binding site was present in all four species: human, *Cavia porcellus*,* H. glaber*, and *Chinchilla chinchilla*. The binding sites found have variations in positions 1, 8, and 9 of PWM, which is consistent with the known ET S binding sites [26, 27, 29]. Thus, we suggest that the identified site is a regulatory element for the hgTER promoter.

The SOX17 binding site was detected solely in the *H. glaber *TER promoter region; it is located ~ 430 nt upstream from the transcription start site. The fact that the SOX17 site is present in *H. glaber *but not in other evolutionary related animals may be an indication of an important difference between these species.

The SOX17 protein contains an HMG_box (Pfam domain high-mobility group box) responsible for the high-affinity binding to non-B-type DNA conformations (kinked or unwound) [30]. The characteristic binding motif in DNA is almost identical for the entire HMG_box family [31, 32]; however, for the transcription factor SOX17 [33, 34] harboring the Sox_C_TAD domain (Pfam: PF12067), the binding sites are largely different from the canonical site – AACAAT [32, 35].

Based on the common architecture for most TER s, we assumed that hgTER also contains the known secondary structure elements [23]. We mainly focused on the rare TER polymorphisms in the important functional elements of the telomerase RN A secondary structure.

**Fig. 1:**
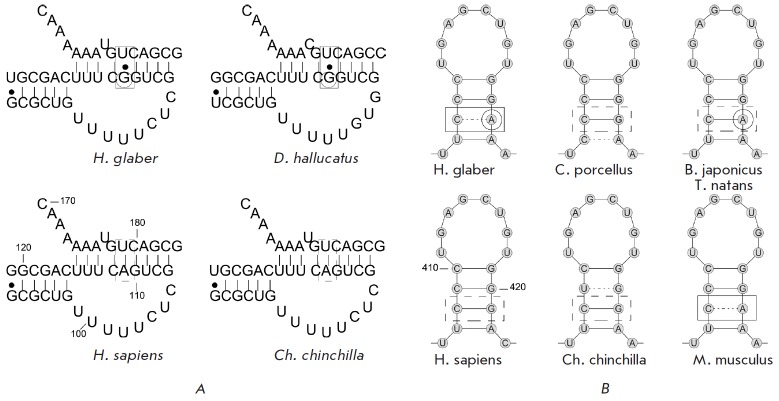
The P2b-p3 and CR7-p8b areas of pseudoknot with the mapped polymorphisms for different species. The polymorphism is marked with a circle; The Watson-Crick base paring is marked with a solid line; non-canonical base pairing is marked with a dashed line. *A *– the P2b-p3 areas of pseudoknot for *H.sapiens*, *Ch. chinchilla*, *H. glaber* and *D.Hallucatus. B *– the CR7-p8b area of the scaRNA TER domain with the mapped polymorphism for *H.sapiens*,* Ch. chinchilla*, *C.porcellus*, *H. glaber*, *M.Musculus*, *Bombina japonic*), and *Typhlonectes natans*

The comparison of hgTER with the TER of *H. glaber’s* closest relatives (guinea pig and chinchilla) and the TER of model organisms (rat, mouse, and humans) revealed a number of differences. Although most changes in *H. glaber *TER were not unique (present in other mammalians) and did not affect the functional elements of TER , we managed to find two rare polymorphisms in the functional region of hgTER . The mapped polymorphisms are shown in *Fig. 1a* and *Fig. 1b*.

The first polymorphism was a A→G replacement in the first loop of pseudoknot P2b-p3 at position 111 (according to the hTER nomenclature). This substitution gives rise to the non-canonical “G–U” pair in the pseudoknot region. Most other TER s have a canonical pair at this position. Moreover, for TER s that have polymorphism A→G111 additional replacement U→C179 takes place that restored the canonical WC pair between bases in 111 and 179 positions (e.g., in *Geomys breviceps* and *Microtus ochrogaster*) [23] The only example of the same G–U pair was revealed in TER s of the *Dasyurus hallucatus *and *Suncus murinus *[23].

The second polymorphism is the replacement G→A in the stem of the scaRN A domain CR 7-p8b at position 421 (according to the hTER nomenclature). This substitution gives rise to the non-canonical “C–A” pair in the p8b stem terminus element at position 421. Most TER s have a canonical base pair at this position. Amphibians (toads and typhlonectes) have G421→A replacement accompanied by C→U transition at position 408, which restores the WC pair [23]. Close relatives – rodents (chinchilla, guinea pig, mice)–have polymorphisms that cause a disturbance in the p8b stem at different positions [23], but this particular substitution at position 421 with the non-canonical pair is unique to *H. glaber*.

## DISCUSSION

Telomerase RN A is a crucial telomerase component, and increased expression of telomerase RN A and te lomerase activity in stem cells or other tissues at different stages of an animal’s development could be an essential reason for its long lifespan. Comparison of the TER gene promoter region and TER in *H. glaber *with available data on other species allowed us to reveal variations both in the promoter region and telomerase RN A structure.

**Fig. 2:**
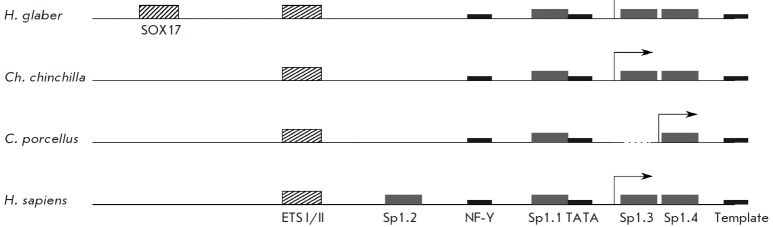
Schematic annotation of the TER promoter areas for humans, guinea pig, mole rat*, *and chinchilla. The reference promoter elements are marked as gray rectangles, and the putative elements predicted in this study are marked as hatched rectangles

An analysis of the region 500 nt upstream of hgTER transcription start side allowed us to identify the regulatory elements known for other organisms [24] and two new ones: the ET S site, which is present in all four model organisms, and the SOX17 site presented only in *H. glaber* (*Fig. 2*). All common elements are located within the ~ 270 nt area, in agreement with the DNase 1 protection data for the human regulatory region [36]. This region contains the TATA box in the proximity of the transcription start site, a NF-Y site with a conserved CC AAT box, SP1 sites, and the newly identified ET S site. In humans, four Sp1 (Sp1.1, Sp1.2, Sp1.3 and Sp1.4) sites were previously identified. An analysis of *H. glaber *and its close relatives revealed that one or more Sp1 sites can be missing in a particular organism. For example, the Sp1.2 site is missing in all rodents (*Fig. 2*) and the Sp1.3 site is also absent in *Cavia porcellus*. In case of humans, the two transcription factors Sp1 and Sp3 can bind to the Sp-sites within the promoter. Sp1 stimulates expression, while Sp3 induces dosedependent repression [36]. The sites adjacent to the CC AAT box from either side (Sp1.1 only for *H. glaber*) are thought to cooperate with NF-Y to mediate positive or negative regulatory effects in humans [37]. The Sp1.3 and Sp1.4 sites adjacent to the transcription start site could also regulate transcription, either positively or negatively, depending on the presence of other proteins that interact with transcription factors [38]. The context of a particular Sp1 site was suggested as essential for the preferential binding of either Sp1 or Sp3 factors, which might influence the TER transcription regulation [38]. Thus, the absence of the Sp1.2 site in rodents may result in differences in the fine regulation of TER transcription via the Sp pathway in rodents, making it more dependent on the particular context of the remaining Sp sites. In case of *H. glaber, *this may have a positive effect on the TER transcription efficiency.

The newly identified ELK4 site is located within the 272 nt area further downstream from the transcription start site. It was found in all the studied species, including humans. ELK4 is a member of the ET S family of transcription factors [29]. For *H. glaber*, the sequence of this site is identical to that of ELK4, but it can also be used by the other members of the ET S family [28]. This factor was identified as a novel target for the androgen receptor-activating cascade. The fact that androgen signaling blockade in the case of prostate cancer reduced telomerase activity indirectly proves that ELK4 participates in the regulation of TER transcription [25].

A SOX17 binding site was found only in *H. glaber*. It is located approximately 430 nt upstream of the gene region, and thus outside of the 272 nt promoter area, which was previously shown to be important for hTER transcription. SOX17 belongs to the family of HMGlike SOX proteins. The SOX17 binding site (ACAAT) is identical for the other members of the SOX proteins, and binding of a particular factor depends on the broader context around the conventional site. SOX17 (SRY-box 17) is a transcription factor involved in the regulation of several developmental processes [39, 40], including endoderm formation, vascular development, and fetal hematopoietic stem cell maintenance.

Sox17 is highly restricted in its expression within the hematopoietic system to fetal hematopoietic stem cells (HSCs) [41]. It has recently been shown that Sox17 expression confers self-renewal potential and fetal stem cell characteristics to adult hematopoietic progenitors [42]. Other SOX proteins are involved in the regulation of various cellular processes. A lack of data does not allow one to propose a particular regulation pathway for the SOX binding site, but the presence of this site is an additional possibility for *H. glaber *to regulate the expression of telomerase RN A and to increase the level of telomerase activity, especially in fetal stem cells. This correlates with earlier studies, where long-living rodent species (such as the *H. glaber *and *Sciurus carolinensis* ) have a higher telomerase activity than mice [43].

Telomerase activity depends not only on the transcriptional level of telomerase components; many other processes are involved, including TER maturation, transport, telomerase assembly, interaction between TER and TERT , etc. Mutations in telomerase RN A can influence these processes.

We found two rare polymorphisms in hgTER : A111→G and G421→A. The A111→G transition in hgTER is located in the stem loop of P2b-p3 pseudoknot. The P2b-P3 pseudoknot (*Fig. 1a*) is highly conserved [23]. The effect of this mutation on the function of telomerase is unknown, but mutations destabilizing the pseudoknot structure affect telomerase activity and lead to aplastic anemia, myelodysplasia, and leukemia in humans [44]. Moreover, mutations that destabilize the pseudoknot structure reduce telomerase activity [45] and lead to dyskeratosis congenita [46]. Polymorphism in this position in other organisms is accompanied by the second mutation that restores the canonical pair. Due to the A→G replacement in *H. glaber*, a non-canonical G–U pair is formed. In contrast to other non-canonical pairs, G–U causes very little distortion to the RN A helix structure [47] and should not have such a severe effect on telomerase as the other ones. The G–U pair in this position is found only in the Asian house shrew and the northern quoll. Life expectancy of the northern quoll is 7 years and about 3 years for shrews [48]. Thus, there is no evident correlation between the existence of the G–U pair in a particular position in the pseudoknot structure and life expectancy.

The polymorphism G→A at position 421 leads to the formation of a C–A non-canonical pair in the p8b stem. Most mammals have the canonical base pair at this position. It should be mentioned that disruption (C→G in C–G) of 408–421 base pairs in humans leads to dyskeratosis congenita [46]. Rodents (chinchilla, guinea pig, mice) have polymorphisms that cause distortion of the p8b stem. The G→A transition in *H. glaber *belongs to the same class of species-dependent variations, but this particular substitution is unique to *H. glaber*. P8b is a part of the CR 7-p8b (H/ACA) domain. CR 7 is required for 3’-end processing, localization, and the stability of hTER [49]. CR 7 contains a conserved Cajal body localization element (CAB box) [50]. The telomerase Cajal body protein 1 (TC AB1) binds to the CAB box [51] and drives hTER to the Cajal body. TC AB1 knockdown prevents telomerase-telomere association and results in telomere shortening [52]. For *H. glaber*, the non-canonical pair “C–A” in the p8b stem loop can improve the interaction between hgTC AB1 and hgTER , make telomerase traffic more effective and thus result in a more efficient telomere elongation*.*

## CONCLUSIONS

Comparison of hgTER and other telomerase RN A genes suggests that both the unique structure of the promoter region and the specific polymorphisms in the functional domains can cause increased expression of the telomerase RN A gene in stem cells, thus reducing replicative senescence and increasing the lifespan. We hope that our finding of a difference in the promoter region of telomerase RN A will inspire other researchers to study these processes using in vivo mouse models.

## Abbreviations

TER: telomerase RNA
hTR: human telomerase RNA
TERT: telomerase catalytic subunit
hTERT: human telomerase catalytic subunit
hgTER: Heterocephalus glaber telomerase RNA

## References

[1] Rando T.A. (2006). Nature.

[2] Van Zant G., Liang Y. (2003). Exp. Hematol..

[3] Rossi D.J., Bryder D., Weissman I.L. (2007). Exp. Gerontol..

[4] Tümpel S., Rudolph K.L. (2012). Ann. N.Y. Acad. Sci..

[5] Blasco M.A. (2007). Nat. Chem. Biol..

[6] Nicholls C., Li H., Wang J.Q., Liu J.P. (2011). Protein Cell..

[7] Jaskelioff M., Muller F.L., Paik J.H., Thomas E., Jiang S., Adams A.C., Sahin E., Kost-Alimova M., Protopopov A., Cadiñanos J. (2011). Nature.

[8] Bernardes de Jesus B., Vera E., Schneeberger K., Tejera A.M., Ayuso E., Bosch F., Blasco M.A. (2012). EMBO Mol. Med..

[9] Collins K. (2011). Curr. Opin. Chem. Biol..

[10] Kelleher C., Teixeira M.T., Forstemann K., Lingner J. (2002). Trends Biochem. Sci..

[11] Chiodi I., Mondello C. (2012). Front. Oncol..

[12] Hiyama E., Hiyama K., Yokoyama T., Shay W.J. (2001). Neoplasia..

[13] Smekalova E.M., Shubernetskaya O.S., Zvereva M.I., Gromenko E.V., Rubtsova M.P., Dontsova O.A. (2012). Biochemistry (Mosc.).

[14] Chiang Y.J., Hemann M.T., Hathcock K.S., Tessarollo L., Feigenbaum L., Hahn W.C., Hodes R.J. (2004). Mol. Cell. Biol..

[15] Garrels W., Kues W.B., Herrmann D., Holler S., Baulain U., Niemann H. (2012). Biol. Reprod.

[16] Kim E.B., Fang X., Fushan A.A., Huang Z., Lobanov A.V., Han L., Marino S.M., Sun X., Turanov A.A., Yang P. (2011). Nature.

[17] Yu C., Li Y., Holmes A., Szafranski K., Faulkes C.G., Coen C.W., Buffenstein R., Platzer M., de Magalhães J.P., Church G.M. (2011). PLoS One..

[18] Gorbunova V., Bozzella M.J., Seluanov A. (2008). Age (Dordr)..

[19] Liang S., Mele J., Wu Y., Buffenstein R. (2010). Aging Cell..

[20] Sandelin A., Alkema W., Engström P., Wasserman W.W., Lenhard B. (2004). Nucleic Acids Research.

[21] Sandelin A., Wasserman W.W., Lenhard B. (2004). Nucleic Acids Research.

[22] Wasserman W.W., Sandelin A. (2004). Nat. Rev. Genet..

[23] Chen J.L., Blasco M.A., Greider C.W. (2000). Cell..

[24] Zhao J.Q., Hoare S.F., McFarlane R., Muir S., Parkinson E.K., Black D.M., Keith W.N. (1998). Oncogene..

[25] Makkonen H., Jääskeläinen T., Pitkänen-Arsiola T., Rytinki M., Waltering K.K., Mättö M., Visakorpi T., Palvimo J.J. (2008). Oncogene..

[26] Shore P., Sharrocks A.D. (1995). Nucleic Acids Research.

[27] Mo Y., Vaessen B., Johnston K., Marmorstein R. (1998). Molecular Cell.

[28] Wei G.H., Badis G., Berger M.F., Kivioja T., Palin K., Enge M., Bonke M., Jolma A., Varjosalo M., Gehrke A.R. (2010). EMBO J..

[29] Sharrocks A.D., Brown A.L., Ling Y., Yates P.R. (1998). Int. J. Biochem. Cell. Biol.

[30] Stros M., Launholt D., Grasser K.D. (2007). Cell. Mol. Life Sci..

[31] Denny P., Swift S., Connor F., Ashworth A. (1992). EMBO J..

[32] Laudet V., Stehelin D., Clevers H. (1993). Nucleic Acids Research.

[33] Dunn T.L., Mynett-Johnson L., Wright E.M., Hosking B.M. (1995). Gene..

[34] Kanai Y., Kanai-Azuma M., Noce T., Saido T.C. (1996). J. Cell. Biol..

[35] Mertin S., McDowall S.G., Harley V.R. (1999). Nucleic Acids Research.

[36] Zhao J.Q., Glasspool R.M., Hoare S.F., Bilsland A., Szatmari I., Keith W.N. (2000). Neoplasia..

[37] Zhao J., Bilsland A., Hoare S.F., Keith W.N. (2003). FEBS Lett..

[38] Li L., Davie J.R. (2010). Ann. Anat..

[39] Séguin C.A., Draper J.S., Nagy A., Rossant J. (2008). J. Cell. Stem Cell..

[40] Patterson E.S., Addis R.C., Shamblott M.J., Gearhart J.D. (2008). Physiol. Genomics..

[41] Kim I., Saunders T.L., Morrison S.J. (2007). Cell..

[42] He S., Kim I., Lim M.S., Morrison S.J. (2011). Genes Dev..

[43] Seluanov A., Chen Z., Hine C., Sasahara T.H. (2007). Aging Cell..

[44] Vulliamy T., Marrone A., Dokal I., Mason P.J. (2002). Lancet..

[45] Chen J.L., Greider C.W. (2005). Proc. Natl. Acad. Sci. USA..

[46] Vulliamy T., Marrone A., Goldman F., Dearlove A., Bessler M., Mason P.J., Dokal I. (2001). Nature.

[47] Leontis N.B., Westhof E. (1998). RN A..

[48] de Magalhães J.P., Costa J. (2009). J. Evol. Biol..

[49] Theimer C.A., Jády B.E., Chim N., Richard P., Breece K.E., Kiss T., Feigon J. (2007). J. Mol. Cell..

[50] Jády B.E., Bertrand E., Kiss T. (2004). J. Cell Biol..

[51] Tycowski K.T., Shu M.D., Kukoyi A., Steitz J.A. (2009). Molecular Cell.

[52] Venteicher A.S., Abreu E.B., Meng Z., McCann K.E., Terns R.M., Veenstra T.D., Terns M.P. (2009). Artandi SE Sci..

